# Rupture of the Tibialis Posterior Tendon With Associated Bimalleolar Ankle Fracture

**DOI:** 10.7759/cureus.31886

**Published:** 2022-11-25

**Authors:** Priyan Magan, Charlene Chin See, Daine Clarke, Ferin Patel, Dhanuja Senn

**Affiliations:** 1 General Surgery, National Health Service (NHS), Wolverhampton, GBR; 2 Trauma and Orthopaedics, Bustamante Hospital for Children, Kingston, JAM; 3 Trauma and Orthopaedics, Faculty of Medical Sciences, The University of the West Indies, Kingston, JAM; 4 General Practice, Russell's Halls, Birmingham, GBR; 5 Plastic Surgery, New Cross Hospital, Wolverhampton, GBR

**Keywords:** bilateral ankle injury, ankle and foot, tibialis posterior tendon, exposed tendon, tendon graft

## Abstract

The acute traumatic rupture of the tibialis posterior tendon in association with closed ankle fractures is rare and often under-recognised. If recognised early, outcomes can be excellent. There are 28 known cases in the literature, and we report two further cases associated with bimalleolar ankle fracture dislocation.

A 49-year-old presented with valgus deformity at the ankle joint and global tenderness following a work injury as a mechanic. A plain radiograph showed a displaced oblique comminuted fracture of the lateral malleolus with valgus angulation at a syndesmosis, with significant talar shift. The patient underwent open reduction and internal fixation with a seven-hole, one-third tubular plate and screws. A 35-year-old involved in a motorcycle collision with a car presented with swollen left ankle and valgus deformity. Plain radiographs revealed bimalleolar fracture subluxation. Closed reduction was unsuccessful and hence direct medial approach demonstrated a complete rupture of the posterior tendon. The medial malleolus was fixed using lag screws and washers. The tendon was repaired using the modified Kessler technique in both cases.

The tibialis posterior plays a significant role in foot and ankle biomechanics due to its broad tendinous insertion. Acute traumatic rupture is rare, as it is protected due to its deep-seated anatomic location within the deep posterior compartment of the leg. Preoperative diagnosis of this injury is challenging and hence this diagnosis is often made intraoperatively. In both cases, there was a retraction of the proximal end beyond incision margins, and this can make tendon rupture difficult to identify intraoperatively as well. Upon identification, assessment of the tendon for degenerative changes was key to deciding upon suitability for primary repair. Despite its rarity, a high index of suspicion should be maintained in fracture dislocation of the ankle joint, especially when the mechanism is known to be pronation-external rotation.

## Introduction

Acute traumatic rupture of the tibialis posterior tendon in association with closed ankle fractures is rare. At best, it is under-recognised or under-reported. The outcome, if identified and repaired in the primary setting, is good to excellent. Missed diagnoses result in loss of subtalar function and ultimately a progressive planovalgus (flat foot) deformity [1]. 

The first case reported in the English literature was in 1980 by Giblin [2]. Since his report, to our knowledge, only 35 have been reported in the English literature (Appendices) [1-31].

We report two cases of acute traumatic tibialis posterior tendon rupture associated with bimalleolar ankle fracture-dislocations identified in the primary setting. In both cases, formal consent was obtained for medical photography.

## Case presentation

Case 1

A 49-year-old male mechanic with no prior history of chronic illnesses was working on a car that slipped off a jack, hitting his right foot and ankle. He experienced immediate pain and swelling to the right ankle, with deformity. He corrected the deformity himself and presented within 24 hours after injury to the Accident and Emergency department where a hemicast was placed. He was referred to the Orthopaedic Outpatient department and seen the following day.

Significant findings on clinical examination were confined to the right ankle. There was significant swelling with a valgus deformity at the level of the ankle joint. There was associated tenderness globally. There were no neurovascular deficits.

**Figure 1:**
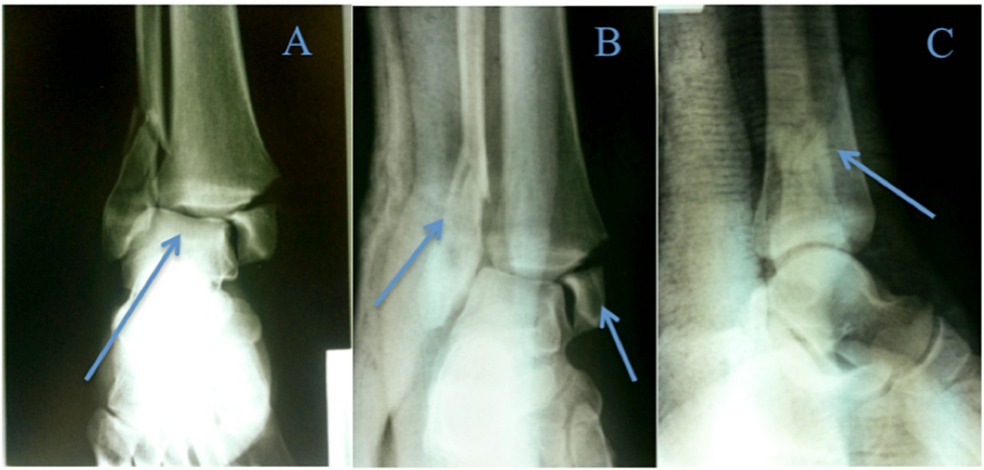
A-C. Orthogonal and mortise views of the right ankle one day prior to presentation, showing a Weber B bimalleolar ankle fracture with lateral talar shift Arrow descriptions: A - talar shift, B - bimalleolar fracture, C - lateral malleolar fracture

Plain radiographs of the right ankle done at presentation revealed a displaced oblique comminuted fracture of the lateral malleolus (Lauge-Hansen pronation-abduction type 3) with 26° of valgus angulation at the level of the syndesmosis and a laterally displaced oblique medial malleolar fracture. There was a significant lateral talar shift (Figures 1A-1C).

**Figure 2:**
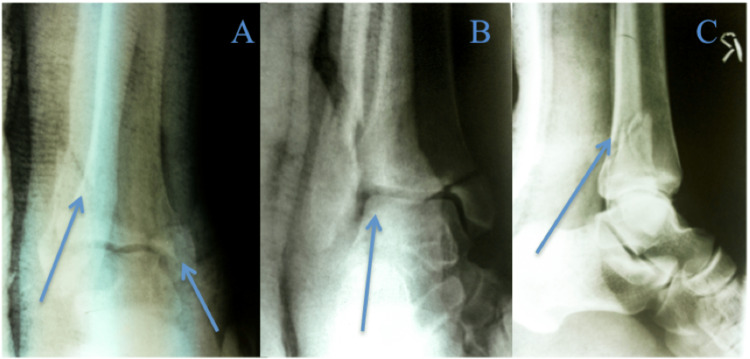
A-C. AP, mortise and lateral views of the right ankle after attempted reduction Arrow descriptions: A - reduction in AP View, B - improvement in talar shift, C - reduction in lateral view AP: anteroposterior

A closed reduction was deemed satisfactory (Figures 2A-2C).

**Figure 3:**
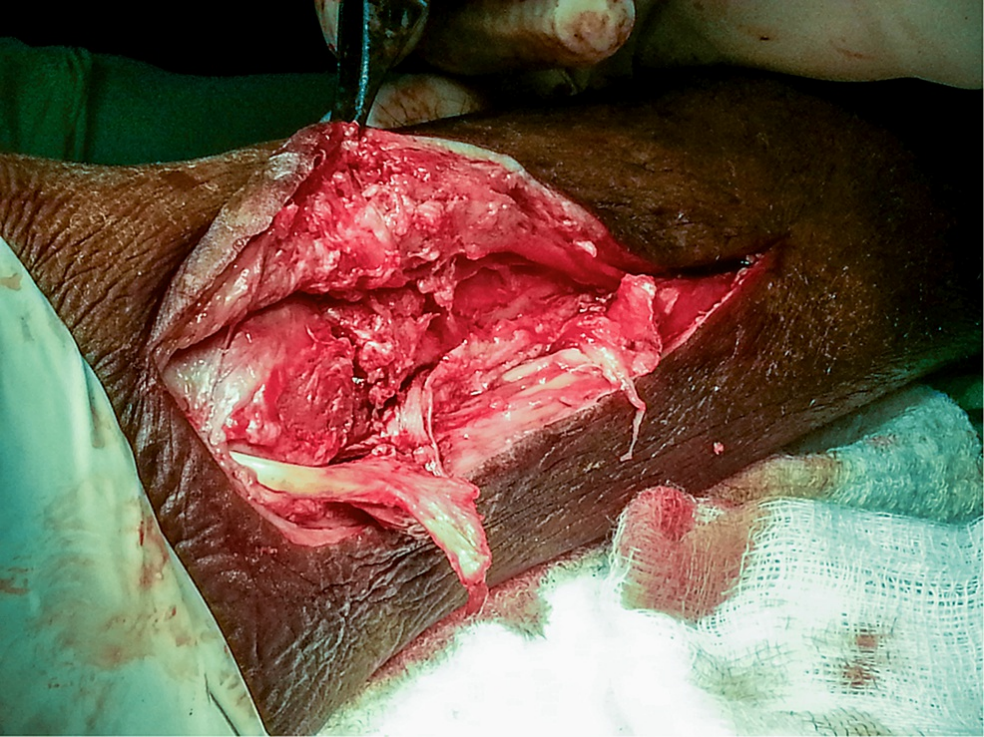
Approach to the medial malleolus showing a transverse medial malleolar fracture and the proximal and distal ends of the tibialis posterior tendon

He was taken to the operating theatre where the lateral malleolus was accessed via a lateral approach. An oblique fracture line with significant comminution was identified. Open reduction and internal fixation were performed with a seven-hole, one-third tubular plate and screws. Cotton’s test was positive and a syndesmotic screw was subsequently placed. A medial curvilinear incision was performed to gain access to the medial malleolus. An oblique fracture was noted with significant lateral displacement of the distal fragment. The tibialis posterior tendon was completely ruptured with the distal stump interposed within the fracture site. The proximal end of the tendon was identified via a proximal extension of the skin incision (Figure 3). No degenerative changes were identified. After manual reduction with the foot inverted, a primary repair was deemed possible.

**Figure 4:**
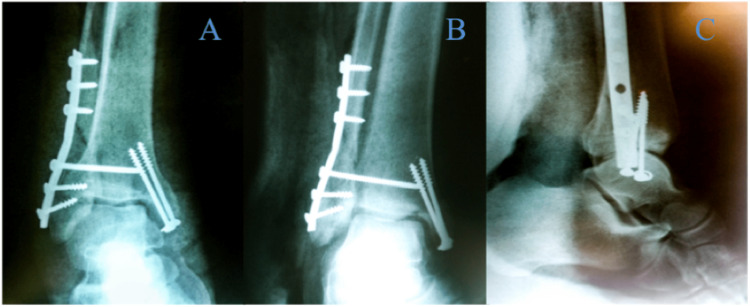
Plain radiographs of the AP, lateral and mortise views of the right ankle post-open reduction internal fixation and posterior tibialis tendon repair A - AP view, B - mortise view, C - lateral view A: anteroposterior

**Figure 5:**
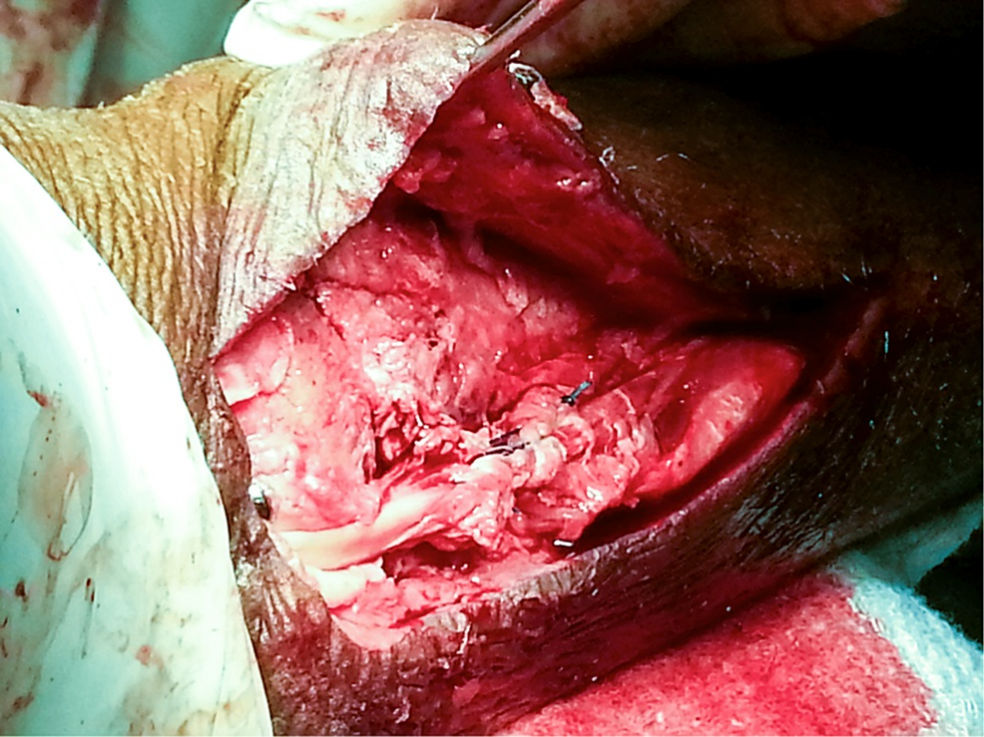
Medial approach to medial malleolus showing repair of right posterior tibialis tendon

The medial malleolar fragment was reduced and two interfragmentary screws with washers were placed (Figures 4A-4C). Subsequently, the tibialis posterior tendon was repaired using a modified Kessler technique (Figure 5). The flexor retinaculum was then also repaired. Post-wound closure, a hemicast was applied with the foot in inversion at the subtalar joint and with slight plantar flexion to decrease the tension on the repaired tendon.

Some two weeks later, he was placed in a full cast with the ankle in neutral, which was maintained until the sixth postoperative week. After radiological confirmation of fracture healing, and with clinical confirmation of tibialis posterior function, he was permitted gradual weight bearing in a controlled ankle movement (CAM) walker with physiotherapy supervision. His postoperative recovery was uneventful.

Case 2

**Figure 6:**
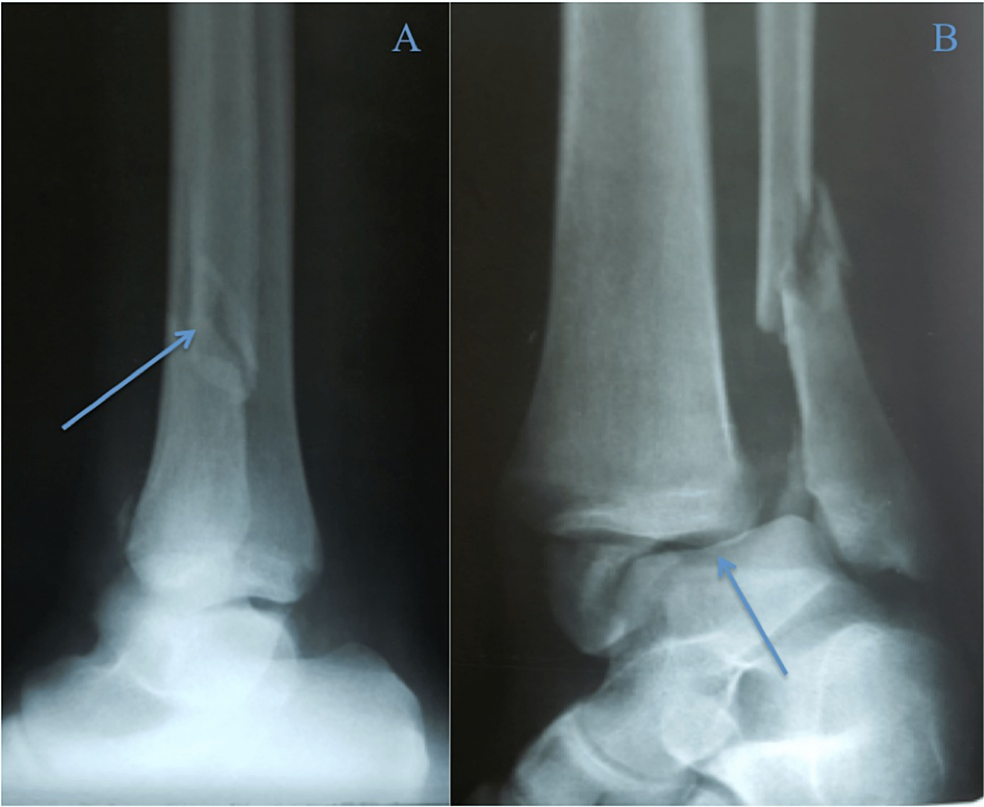
Plain radiographs (lateral and mortise views) Arrow descriptions: A - fracture site seen in lateral view, B - subluxation seen in mortise view

A 35-year-old, male, helmetless rider lost control of his motorcycle and collided with a car. He was thrown from the motorcycle and sustained trauma to his right ankle. There were no stigmata of head injury, and his only complaint was of pain, deformity to his right ankle and an inability to weight bear. Examination findings in the emergency department revealed a globally swollen left ankle with a valgus deformity. A 5x3 cm abrasion on the medial aspect of the left ankle was also noted. He had no distal neurovascular deficit. Plain radiographs of the ankle revealed a bimalleolar fracture subluxation (Figure 6).

**Figure 7:**
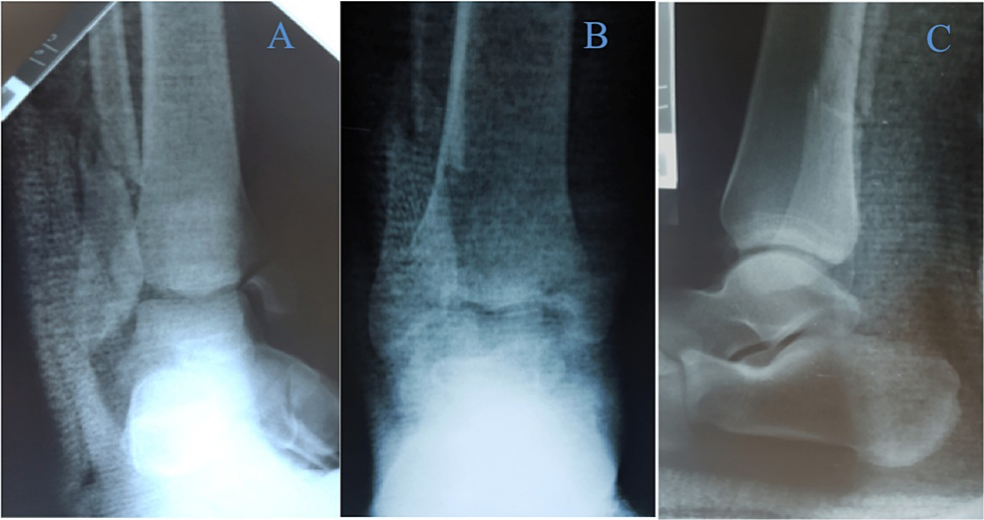
Plain radiographs post-reduction (mortise, AP and lateral views) A - mortise view, B - AP view, C - lateral view AP: anteroposterior

Manipulation under sedation was subsequently undertaken and a dorsal hemicast was placed (Figure 7). 

**Figure 8:**
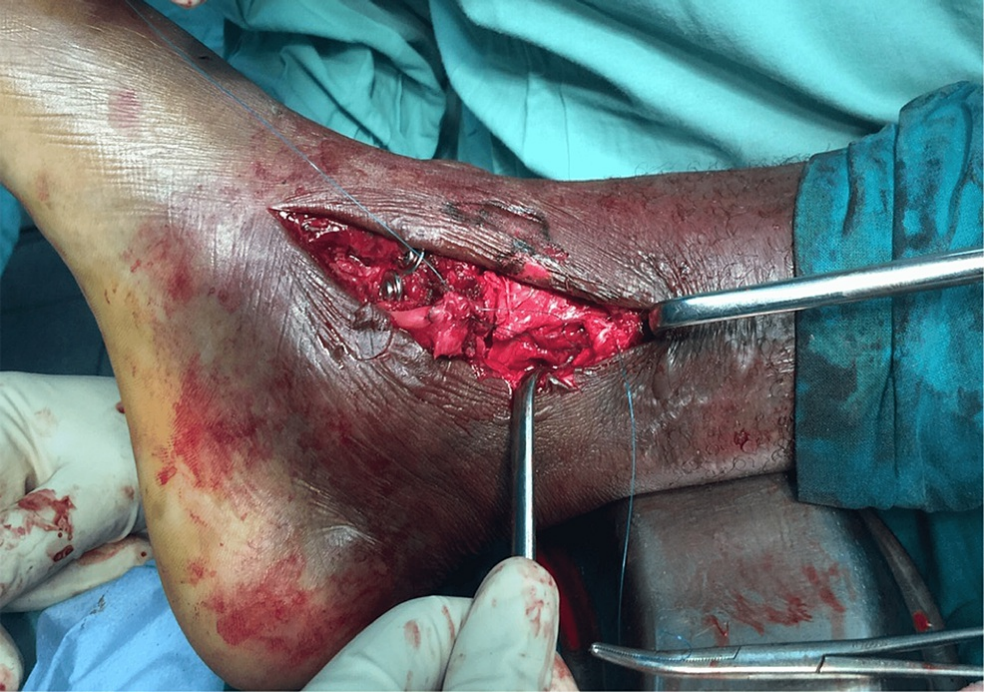
Intraoperative image showing the ruptured tibialis posterior tendon

**Figure 9:**
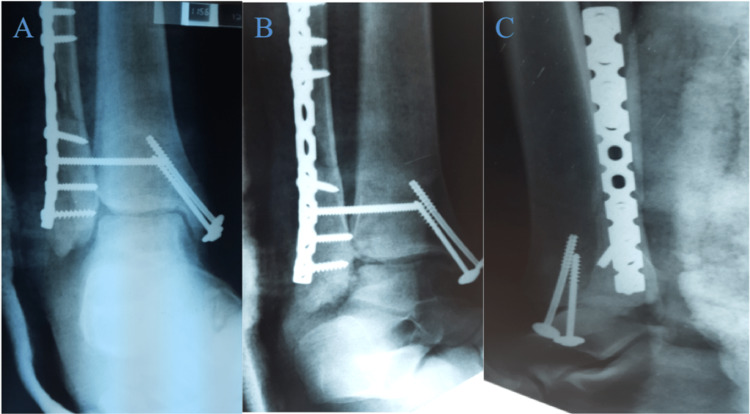
Post-operative radiographs (AP, mortise and lateral views) A - AP view, B - mortise view, C - lateral view AP: anteroposterior

Following this initial management to ensure that any intervention would not compromise the soft tissue envelope, five days later, he underwent open reduction and internal fixation. Intraoperatively after reduction and fixation of the lateral malleolus, closed reduction of the medial malleolar fracture was attempted but was unsuccessful. A direct medial approach to the ankle joint was performed. Upon exploration, the tibialis posterior tendon was found to be completely ruptured from the distal end interposed within the fracture site (Figure 8). There were no signs of degenerative changes. The medial malleolus was reduced and fixation was performed with 4 mm lag screws and washers. The tibialis posterior tendon edges were debrided and primary repair was undertaken using 3-0 prolene via a modified Kessler technique. The ankle was then splinted in 20 degrees plantar flexion and inversion. Post-operative radiographs were satisfactory (Figure 9).

## Discussion

The tibialis posterior plays a significant role in foot and ankle biomechanics due to its broad tendinous insertion [1-2]. Inclusive in this role is the maintenance of the medial longitudinal arch, subtalar joint stabilisation during gait, inversion of the subtalar joint and flexion of the ankle joint.

The tibialis posterior acts as the initiator of hind foot inversion during the stance phase of the gait cycle. As this occurs, the calcaneocuboid and talonavicular joints lose their parallelism, converting the foot into a rigid lever to facilitate heel rise and toe-off. As the hind foot is inverted, the axis of the gastrocnemius-soleus complex comes to lie medial to the axis of rotation of the subtalar joint and locks the joint as well as the transverse tarsal joint. The rigid lever created propels the foot during the final stage of the single-limb stance of gait [3]. Dysfunction of the tibialis posterior results in failure to create this rigid lever and the forces are transmitted to the medial longitudinal arch supporters resulting in attenuation over time. The end result of the failure of the tibialis posterior tendon is a progressive flat foot deformity and its sequelae, including chronic foot pain.

Tibialis posterior tendon rupture is usually due to inflammatory or degenerative causes but rarely acute traumatic causes [3-8]. This is mostly due to its anatomic course. It is protected from traumatic injury from external implements due to its deep-seated anatomic location within the deep posterior compartment of the leg. It originates from the posterior lateral aspect of the tibia, the posterior medial aspect of the fibula and the intervening interosseous membrane. Distally, it passes deep into the flexor retinaculum and lies in a groove behind the medial malleolus under the coverage of the flexor retinaculum. The tendon courses superficial to the deltoid ligament and then deep to the plantar calcaneal navicular ligament on its way to its main insertion in the navicular bone. As it passes in relation to the medial malleolus, it makes a sharp bend and changes direction toward its insertion. The medial malleolus acts as a pulley, allowing the gliding tendon to change its direction of pull [1]. There is a relatively avascular segment commencing approximately 1-1.5 cm distal to the medial malleolus and extending another centimetre [9-10]. This is the location for most traumatic tibialis posterior tendon ruptures and usually occurs secondary to pronation-external rotation mechanisms [11-12].

The preoperative diagnosis of this injury is challenging because the pain from the concomitant ankle fracture makes it difficult to examine for posterior tibialis tendon function [2-8]. Preoperative diagnosis of a dislocated, intact posterior tibialis tendon has been made reportedly by palpation of the cordlike structure over the medial malleolus. The diagnosis of a traumatic rupture on the other hand is usually made intraoperatively or retrospectively. The diagnosis, however, should be entertained in all irreducible ankle fractures. Radiographic clues have also been suggested. Stein reported two cases in which metaphyseal bone flakes were noted and their absence in his subsequent review of 30 cases of medial malleolar fractures without associated tibialis posterior tendon rupture. He suggested that this feature could be diagnostic [5].

Intraoperatively, rupture of the tendon can easily go unidentified because of retraction of the tendon proximally beyond standard proximal incision boundaries and retraction of the distal end under the cover of the flexor retinaculum [4,6]. In both cases, there was a retraction of the proximal end beyond the incision margin. Once identified, the tendon should be assessed for degenerative changes. In the absence of degenerative changes, early primary repair should be undertaken [3,8]. In cases in which there is tendon loss or large gaps in flexor digitorum longus (FDL) or flexor hallucis longus (FHL), a transfer may be utilised. There is a size discrepancy between FDL and TP, however, the location and close proximity facilitate an easier transfer. Patients are still able to perform satisfactory single heel raises post-transfer [1].

## Conclusions

The tibialis posterior tendon plays an important role in gait. Rupture of the tibialis posterior tendon in association with closed ankle fractures is a rare entity. Despite its rarity, a high index of suspicion should be maintained in fracture dislocation of the ankle joint, especially when the mechanism is known to be pronation-external rotation. Identification and primary repair offer the best outcome.

## Appendices

**Table 1:** Thirty-five known cases of acute traumatic tibialis posterior tendon rupture in current literature

Authors	Title	Journal	Year	Number of cases
Giblin, M. M.	Ruptured tibialis posterior tendon associated with a closed fracture medial malleolus [2]	Aust N Z J Surg	1980	1
Kelbel, M.; Jardon, O. M.	Rupture of tibialis posterior tendon in a closed ankle fracture [3]	J Trauma	1982	1
De Zwart, D. F.; Davidson, J. S. A.	Rupture of the posterior tibial tendon associated with fractures of the ankle [4]	J Bone Joint Surg Am	1983	2
Stein, R. E.	Rupture of the posterior tibial tendon in closed ankle fractures. Possible prognostic value of a medial bone flake: report of two cases [5]	J Bone Joint Surg Am	1985	2
Schaffer, J. J.; Lock, T. R.; Salciccioli, G. G.	Posterior tibial tendon rupture in pronation external rotation ankle fractures [6]	J Trauma	1987	2
Soballe, K.; Kjersgaard-Andersen, P.	Ruptured tibialis posterior tendon in a closed ankle fracture [13]	Clin Orthop	1988	2
Monto, R. R.; Moorman, C. T.; Mallon, W. J.; Nunley, J. A.	Rupture of the posterior tibial tendon associated with ankle fracture [7]	Foot Ankle	1991	1
Burton, PD; Page BJ 2^nd^.	Fracture of the neck of the talus associated with a trimalleolar ankle fracture and ruptured tibialis posterior tendon [14]	J Orthop Trauma	1992	1
Ebraheim, N. A.; Wong, F. Y.	Simultaneous fracture of the ankle and talus associated with ruptured tibialis posterior tendon [15]	J Orthop Am	1995	1
Rosenberg, GA; Sferra, JJ	Checkrein Deformity–An Unusual Complication Associated with a Closed Salter-Harris Type II Ankle Fracture: A Case Report [16]	Foot & Ankle International	1999	1
Mallick, S.; Faleme, A.	Traumatic rupture of the tibialis posterior tendon after closed ankle fractures: a report of two cases [17]	Eur J Orthop Surg	2001	2
Sharma, H.; Meredith, A. D.	Concomitant traumatic rupture of the tibialis posterior tendon with a closed complex ankle fracture. An uncommon injury, which can easily be overlooked [18]	The Foot	2004	1
Ceccarelli, F.; Faldini, C.; Pagkrati, S.; Giannini, S.	Rupture of the tibialis posterior tendon in a closed ankle fracture: a case report [8]	Chirurgia Degli Organi di Movimento	2008	1
Penney, K. E.; Wiener, B. D.; Magill, R. M.	Traumatic rupture of the tibialis posterior tendon after ankle fracture: a case report [19]	Am J Orthop (Belle Mead NJ)	2000	1
West, M. A.; Sangani, C.; Toh, E.	Tibialis posterior tendon rupture associated with a closed medial malleolar fracture: a case report and literature review [9]	J Foot Ankle Surg	2010	1
Jarvis, H. C.; Cannada, L. K.	Acute tibialis posterior tendon rupture associated with a distal tibial fracture [1]	Orthopedics	2012	1
Al Khudairy, A.; Zafar, M. M.; Padinjarathaia, B. A.	The unexpected with ankle fracture: traumatic tibialis posterior tendon dislocation: a case report and literature review [20]	Foot Ankle Spec	2013	1
Phillips, S. L.; Williams, D.; Jeyaseeian, L.; Bryce, E.; Alyas, F.; Vemulapalli, K.	Unilateral adolescent pes planus after a bimalleolar ankle fracture: a case report [21]	J Foot Ankle Surg	2015	1
KarabilaM.A, AzouzM., Mhamdi Y., Hmouri I., Kharmaz M., Bardouni A., Lahlou A., MahfoudM., Berrada M.S.	Traumatic rupture of the posterior tibial tendon occurring during a closed fracture of the ankle: report of a case. [22]	Pan Afr Med J	2015	1
Krishna S.V., Pilar A., Pai S.N., Issac T.	Traumatic Rupture of Posterior Tibial Tendon Following Closed Supination-Adduction Ankle Fracture: A Case Report [23]	JBJS Case Connect	2016	1
Formica, M.; Santolini, F.; Alessio-Mazzola, M.; Repetto, I.; Andretta, A.; Stella, M.	Closed medial malleolar multifragment fracture with a posterior tibialis tendon rupture: a case report and review of the literature [24]	J Foot Ankle Surg	2016	1
Park, J.	Partial rupture of the tibialis posterior tendon associated with a closed medial malleolar fracture (A case report) [25]	Journal of the American Podiatric Medical Association	2016	1
Jasqui-Remba S, Rodriguez-Corlay RE.	Muscular tendinous junction rupture of the posterior tibial tendon after closed bimalleolar ankle fracture [26]	BMJ Case Rep	2016	1
Bernstein D.T., Harris J.D., Cosculluela P.E., Varner K.E.	Acute tibialis posterior tendon rupture with pronation-type ankle fractures [27]	Orthopedics	2016	3
Wardell R.M., Hanselman A.E., Daffner S.D., SantrockR.D.	Posterior tibialis tendon rupture in a closed bimalleolar-equivalent ankle fracture: case report [28]	Foot Ankle Spec	2017	1
Vosoughi A.R., Ravanbod H., Gilheany M., Erfani M.A., Mozaffarian K.	Posterior tibialis tendon rupture associated with closed medial malleolus fracture and avulsion of anterior talofibular ligament: A case report and review of the literature [29]	Hong Kong Journal of Emergency Medicine	2018	1
Cataldi, C. Bacci, N. Colasanti, G. Moreschini, F. Muratori, F. Mondanelli, N. Giannotti, S.	Posterior tibial tendon rupture associated with anterolateral distal tibial and medial malleolar fracture and a novel pattern of tibiofibular syndesmotic injury: a case report and review of the literature [30]	J Foot Ankle Surg	2020	1
Talebi S., Sheibani S., Ghaffari S., Ghadiri A.	Posterior tibialis tendon rupture concomitant with a closed medial malleolar fracture: A case report and literature review [31]	Journal of Orthopaedics, Trauma and Rehabilitation	2021	1
