# Correction: Rupture of the Tibialis Posterior Tendon With Associated Bimalleolar Ankle Fracture

**DOI:** 10.7759/cureus.c95

**Published:** 2023-01-15

**Authors:** Priyan Magan, Charlene Chin See, Daine Clarke, Ferin Patel, Dhanuja Senn

**Affiliations:** 1 General Surgery, National Health Service (NHS), Wolverhampton, GBR; 2 Trauma and Orthopaedics, Bustamante Hospital for Children, Kingston, JAM; 3 Trauma and Orthopaedics, Faculty of Medical Sciences, The University of the West Indies, Kingston, JAM; 4 General Practice, Russell's Halls, Birmingham, GBR; 5 Plastic Surgery, New Cross Hospital, Wolverhampton, GBR

This article has been corrected at the request of the authors to add author Charlene Chin See, who was erroneously omitted from the original submitted and published article. The updated author list is as follows:

Priyan Magan

Charlene Chin See

Daine Clarke

Ferin Patel

Dhanuja Senn

Additionally, author Daine Clarke’s affiliation has been corrected from Orthopaedic Surgery, Royal Derby Hospital to Trauma and Orthopaedics, Faculty of Medical Sciences, The University of the West Indies.

The authors deeply regret that these errors were not identified and addressed prior to publication.

 

